# Association Between Diet and Seborrheic Dermatitis: A Case-Control Study

**DOI:** 10.7759/cureus.48782

**Published:** 2023-11-14

**Authors:** Malak Alshaebi, Lina Zahed, Majed Osaylan, Sanaa Sulaimani, Abdulrahman Albahlool, Mohammed H Abduljabbar, Jehad Hariri

**Affiliations:** 1 Dermatology, King Abdulaziz University Hospital, Jeddah, SAU; 2 Clinical Nutrition, King Abdulaziz University Hospital, Jeddah, SAU; 3 Medicine, King Abdulaziz University Hospital, Jeddah, SAU; 4 Medicine, Dr. Soliman Fakeeh Hospital, Jeddah, SAU; 5 Dermatology, King Fahad General Hospital, Jeddah, SAU

**Keywords:** diet and dermatology, food frequency questionnaire, food and nutrition, diet, seborrheic dermatitis

## Abstract

Introduction

Seborrheic dermatitis (SD) is a chronic, inflammatory papulosquamous skin disease. The symptoms and signs of SD are typically managed by topical ketoconazole and topical corticosteroids. However, they don't provide a cure for SD, which explains the disease's chronic nature. With this study, we aimed to identify specific dietary modifications that can be implemented as an adjunctive to traditional treatment of SD.

Methods

A case-control retrospective study. Data was obtained from medical records of patients diagnosed with SD. Patients were invited to participate in an online self-reported questionnaire, and dietary intake was assessed using a validated food frequency questionnaire. The controls were collected by distributing the same questionnaire to healthy adult residents living in Jeddah via social media.

Results

Two hundred sixty-seven participants were included in the study, 59 of whom were SD patients. Participants who reported consuming the following food types once daily had a higher percentage of SD compared to those without SD: a slice of white bread (p*=*0.002), a cup of rice or pasta (p<0.001), non-acidic fruits (p*=*0.014), leafy green vegetables (p=0.007), other types of vegetables (p=0.001), roasted or fried nuts (p=0.047), raw nuts (p=0.022) and a cup of coffee (p=0.041). When asked about their impression of what food types triggered or relieved their disease, 29 (49.2%) of the diseased participants reported no flare-ups with any kind of food. On the other hand, the following food types were commonly reported to be associated with SD exacerbation: spicy food (16.9%), sweets (16.9%), fried food (13.5%), dairy products (11.9%), and citrus fruits (10.2%). In contrast, citrus fruits, leafy green vegetables (8.5% for each), and the other types of vegetables (6.8%) were frequently observed with SD improvement.

Conclusion

Several dietary factors have been associated with SD in our cohort. Thus, our findings could offer new insights into the application of adjuvant dietary measures that might play a role in the improvement of SD symptoms and potentially enhance therapeutic outcomes.

## Introduction

Seborrheic dermatitis (SD) is a chronic, inflammatory papulosquamous skin disease primarily affecting sebum-rich areas such as the face, anterior chest, and scalp. SD usually presents in patients as flaky, greasy, erythematous patches along the nasolabial fold, postauricular area, and forehead. A clinical diagnosis can be made easily based on the distribution and presentation of symptoms. A previous retrospective cross-sectional study estimated that almost 5% of the population is affected by this relapsing condition [1]. In Jeddah, a coastal city in the west of Saudi Arabia, SD encompassed 2.2% of skin diseases, as concluded by a retrospective prevalence study done in King Abdulaziz University Hospital [2]. 

The underlying pathophysiology of SD is not well understood. However, many studies have identified several predisposing factors, such as *Malassezia* yeast colonization, sebaceous gland activity, and individual susceptibility. *Malassezia* are commensal yeast that thrives in sebaceous areas high in lipids. They metabolize the triglycerides found in sebum by releasing lipase. The resulting byproducts, free fatty acids, and reactive oxygen species, not only cause injury to the surrounding cells but also elicit the immune system to generate inflammatory cytokines such as IL-8 and TNF-α which in turn drives keratinocytes to proliferate, causing the characteristic scales seen in SD. Disruption of the epidermal barrier integrity occurs due to a decrease in ceramides and structural keratin (K1, K10, and K11), ultimately making erythematous patches appear. Other predisposing factors include HIV, in which SD can be seen in its severest form due to a dysregulated immune response. Parkinson's disease is another risk factor explained by a hyperactive parasympathetic system and immobility causing sebum accumulation [3].

SD has a biphasic incidence. The first peak prevalence of SD occurs in infancy, causing what's known to be as a cradle cap due to the effect of maternal androgens stimulating the growth of sebaceous glands. The second peak prevalence is seen in adults in their third and fourth decade, mostly in males, due to an increase in sebaceous glands activity by androgens. The latter is taking a more chronic relapsing course and is the main focus of this article.

The treatment of SD is based on the understanding of the pathophysiology. Antifungals such as topical ketoconazole are the mainstay of treatment for SD as they target the *Malassezia* yeast and limit their growth. Topical corticosteroids can be used in combination with antifungal therapy, but long-term use should be avoided due to their adverse effects, such as epidermal atrophy, striae, or telangiectasia [4]. These treatment options typically manage the signs and symptoms of SD. However, they don't provide a cure for SD, which explains the chronicity of the disease and the recurrent flare-ups. Therefore, other treatment options, such as dietary and lifestyle modifications, should be identified to help in the management of SD and prevent recurrent episodes, thereby decreasing the disease burden.

There has been extensive research on dermatological diseases and their relationship with diet. Certainly, nutritional deficiencies can cause a myriad of symptoms and diseases, such as scurvy or pellagra, but the main focus of this research is to study dietary intake rather than nutritional deficiency as an etiological agent for SD. Currently, only food allergies and dermatitis herpetiformis have been proven to be caused by the intake of certain types of food. In recent years, however, some studies have emerged, proving that diet can also affect primary dermatological diseases. For example, a review article concluded that a high glycemic load diet has been strongly associated with acne vulgaris as well as dairy products, but to a lesser extent [5]. In addition, a randomized controlled trial demonstrated an improvement of acne lesions clinically as well as a reduction in the size of sebaceous glands by histopathological examination after modifying the patients' diet to a low glycemic load diet for 10 weeks [6]. The mechanism behind this association is most likely due to the sudden increase of serum insulin and insulin-like growth factor-1 (IGF-1) after consuming easily absorbed carbohydrates, such as white bread. Thereby motivating androgen synthesis, increasing androgen bioavailability, and eventually stimulating sebum production, which all play a vital role in the pathogenesis of acne. Furthermore, a cross-sectional study published in 2016 assessed the dietary habits of 17,497 adults and found a significant association between instant noodles, meat, processed food, and atopic dermatitis (p<0.001). The mechanism behind this finding is yet to be confirmed, but food additives such as monosodium glutamate are thought to contribute to the severity of atopic dermatitis by acting as a pseudo-allergen [7]. 

When examining the association between SD and dietary habits, only a few articles have been published, all of which produced inconsistent findings and contradictory results. In Saudi Arabia, no research has ever been conducted on the relationship between diet and SD. With this study, we aim to fill this gap in the literature by investigating the dietary habits of patients diagnosed with SD in comparison to healthy controls in Jeddah, Saudi Arabia. We suggest a different route of controlling the burdensome symptoms of SD by considering nutrition as a risk factor or possibly a disease aggravator. Our objective is to determine if there is a relationship between diet and SD and, if so, what specific type of food intake increases the risk or severity amongst those diagnosed with the disease. The end goal is to identify specific dietary modifications that can be implemented as an adjunctive to traditional treatment of SD in everyday clinical practice.

## Materials and methods

Study design

This is a case-control, non-interventional retrospective study. After obtaining institutional review board approval, data was collected from 2020 to 2023 by obtaining electronic medical records of those diagnosed with SD from the dermatology department of two governmental hospitals and one private hospital in the city of Jeddah. After obtaining their consent, patients were instructed to fill out a survey that included questions about their diet as well as their disease. Dietary intake was assessed using a food frequency questionnaire (FFQ) based on a published validated Saudi FFQ [8]. The controls were collected by distributing the questionnaire via social media. Participants were instructed to complete the questionnaire based on their dietary recall over the last year.

Participants

In this study, we included patients who are diagnosed with SD based on hospital medical records and are adults above the age of 18. Our exclusion criteria included having a chronic condition like diabetes, hypertension, or thyroid disease. We also excluded those who are smokers, pregnant or lactating, and who have been diagnosed with an eating disorder. For the controls, we included healthy adults who are residents of Jeddah. The control group was asked in the survey whether they were ever diagnosed with seborrheic dermatitis by a dermatologist. Those who answered 'No' were included, and the same exclusion criteria were applied to the controls. 

Sample size and data collection

Sample size calculation was based on the following assumptions: 80% power, 5% significance level, and a case-to-control ratio of 1:1. Since the rate of exposure to seborrheic dermatitis among the control group was 4.6%, as shown in a previous epidemiological study [9]. The required sample was estimated at 414 subjects (207 subjects in each group), but we were not able to reach that number due to poor response. The Epi Info^TM^ software (version 7.2.5.0) was used for the calculation of the unmatched case-control investigations. Electronic medical records of patients diagnosed with SD were reviewed in three hospitals to obtain their contact numbers. More than 1000 patients were contacted over three years via text message using WhatsApp to fill out the questionnaire after obtaining their consent. The same questionnaire was distributed among the residents of Jeddah via WhatsApp. The sample size is ultimately 267 participants after exclusion; 59 of them were within the SD cohort.

Statistical analysis

Data analysis was performed using RStudio (R version 3.4.0; R Foundation, Vienna, Austria). Categorical data were presented as frequencies and percentages. Association analyses were conducted using a Chi-squared test or a Fisher's exact test as appropriate. A multiple-response analysis was performed for variables with multiple available responses. A p-value of <0.05 indicated statistical significance.

## Results

Demographic characteristics by the status of being diagnosed with seborrheic dermatitis

Initially, we received 360 responses on the online platform. However, we excluded 10 records of patients aged <18 years to not include cases with infantile seborrheic dermatitis, and 83 records of those with chronic conditions due to their possibility of dietary limitations and to avoid confounding factors. Therefore, we analyzed the data of 267 participants in the current study. A total of 59 respondents had previously been diagnosed with SD, representing 22.1% of the sample. The majority of participants were female, 163 (61.0%), and the most common age group was between 18 and 30 years, accounting for 36.3% (n=97) of the total. The vast majority of participants were Saudi nationals 257 (96.3%). In terms of BMI, the most prevalent category was overweight, comprising 40.3% (n=106) of the participants. Regarding educational level, the majority of participants had a bachelor's degree 180 (67.4%). The primary residence city for the participants was Jeddah 221 (82.8%). In terms of physical exercise, nearly half of the participants did not engage in sports 129 (48.3%). Moreover, the majority of participants were not following a specific diet 229 (85.8%) and were not currently taking weight-loss medications or therapies 261 (97.8%). More details about the demographic characteristics and clinical history are presented in Table 1. Age was significantly associated with SD, with a p-value of <0.001. Participants aged 18 to <30 years had a higher proportion of "Yes" responses, 32 (54.2%) compared to other age groups. Conversely, participants aged 45 to <60 years had a lower proportion of "Yes" responses 5 (8.5%). Gender also showed a significant association with a P-value of 0.034. Among the "Yes" responses, a slightly higher percentage of males, 30 (50.8%), reported the condition compared to females 29 (49.2%).

**Table 1:** Demographic characteristics of the respondents by their status of being diagnosed with seborrheic dermatitis

Characteristic	Missing	Overall, N=267	No, n=208	Yes, n=59	p-value
Age	0 (0%)				<0.001
18 to <30		97 (36.3%)	65 (31.3%)	32 (54.2%)	
30 to <45		88 (33.0%)	67 (32.2%)	21 (35.6%)	
45 to <60		77 (28.8%)	72 (34.6%)	5 (8.5%)	
60 or more		5 (1.9%)	4 (1.9%)	1 (1.7%)	
Gender	0 (0%)				0.034
Male		104 (39.0%)	74 (35.6%)	30 (50.8%)	
Female		163 (61.0%)	134 (64.4%)	29 (49.2%)	
Nationality	0 (0%)				0.124
Saudi		257 (96.3%)	198 (95.2%)	59 (100.0%)	
Non-Saudi		10 (3.7%)	10 (4.8%)	0 (0.0%)	
BMI (kg/m2)	4 (1.5%)				0.760
Underweight <18.5		13 (4.9%)	9 (4.4%)	4 (6.9%)	
Normal weight 18.5 to <25		76 (28.9%)	58 (28.3%)	18 (31.0%)	
Overweight 25.0 to <30		106 (40.3%)	83 (40.5%)	23 (39.7%)	
Obese ≥ 30.0		68 (25.9%)	55 (26.8%)	13 (22.4%)	
Educational level	0 (0%)				0.227
Uneducated		2 (0.7%)	1 (0.5%)	1 (1.7%)	
Primary		0 (0.0%)	0 (0.0%)	0 (0.0%)	
Middle		1 (0.4%)	1 (0.5%)	0 (0.0%)	
Secondary/diploma		52 (19.5%)	39 (18.8%)	13 (22.0%)	
Bachelor's degree		180 (67.4%)	138 (66.3%)	42 (71.2%)	
Postgraduate		32 (12.0%)	29 (13.9%)	3 (5.1%)	
Residence city	0 (0%)				<0.001
Jeddah		221 (82.8%)	162 (77.9%)	59 (100.0%)	
Other		46 (17.2%)	46 (22.1%)	0 (0.0%)	
Physical exercise	0 (0%)				0.801
I do not do sports		129 (48.3%)	101 (48.6%)	28 (47.5%)	
2-3 times a week		104 (39.0%)	82 (39.4%)	22 (37.3%)	
4-6 times a week		34 (12.7%)	25 (12.0%)	9 (15.3%)	
Following a specific diet	0 (0%)				0.272
No		229 (85.8%)	181 (87.0%)	48 (81.4%)	
Yes		38 (14.2%)	27 (13.0%)	11 (18.6%)	
Currently taking weight-loss medications or therapies	0 (0%)				>0.999
No		261 (97.8%)	203 (97.6%)	58 (98.3%)	
Yes		6 (2.2%)	5 (2.4%)	1 (1.7%)	
Ever diagnosed with an eating disorder before	0 (0%)				>0.999
No		267 (100.0%)	208 (100.0%)	59 (100.0%)	
Yes		0 (0.0%)	0 (0.0%)	0 (0.0%)	

Analysis of the frequency of food consumption

**Figure 1:**
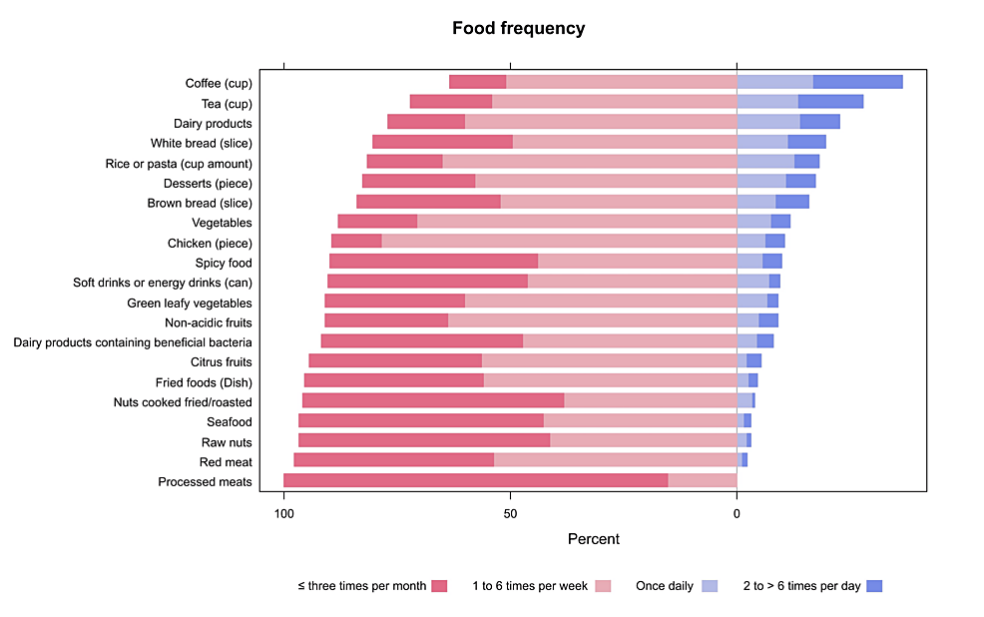
Proportions of participants' responses to the food frequency questionnaire

Internal consistency analysis of the food frequency questionnaire showed a high level of internal consistency (Cronbach's alpha =0.829). The detailed responses to the questionnaire are depicted in Figure 1 and demonstrated in Table 2. Regarding the association between consumption of food types and SD, results showed that participants who reported consuming the following food types once daily had a higher percentage of SD compared to those without SD: a slice of white bread (22.0% n=13 vs. 8.2% n=17, respectively, p=0.002), a cup of rice or pasta (35.6% n=21 vs. 10.6% n=22, respectively, p<0.001), non-acidic fruits (15.3% n=9 vs. 3.4% n=7, respectively, p=0.014), leafy green vegetables (15.3% n=22 vs. 4.8% n=10, respectively, p=0.007), other types of vegetables (22.0% n=13 vs. 7.2% n=15, respectively, p=0.001), roasted or fried nuts (8.5% n=5 vs. 1.4% n=3, respectively, p=0.047), raw nuts (10.2% n=6 vs. 1.9% n=4, respectively, p=0.022) and a cup of coffee (23.7% n=14 vs. 18.8% n=39, respectively, p=0.041) Further details are presented in Table 2.

**Table 2:** Results of participants' responses to the food frequency questionnaire

Characteristic	Overall, N=267	Seborrheic dermatitis		p-value
		No, n=208	Yes, n=59	
Red meat				0.067
≤3 times per month	118 (44.2%)	95 (45.7%)	23 (39.0%)	
1 to 6 times per week	144 (53.9%)	111 (53.4%)	33 (55.9%)	
Once daily	4 (1.5%)	1 (0.5%)	3 (5.1%)	
2 to > 6 times per day	1 (0.4%)	1 (0.5%)	0 (0.0%)	
Chicken (piece)				0.343
≤3 times per month	25 (9.4%)	19 (9.1%)	6 (10.2%)	
1 to 6 times per week	205 (76.8%)	164 (78.8%)	41 (69.5%)	
Once daily	21 (7.9%)	14 (6.7%)	7 (11.9%)	
2 to > 6 times per day	16 (6.0%)	11 (5.3%)	5 (8.5%)	
Seafood				0.107
≤3 times per month	154 (57.7%)	114 (54.8%)	40 (67.8%)	
1 to 6 times per week	104 (39.0%)	88 (42.3%)	16 (27.1%)	
Once daily	6 (2.2%)	4 (1.9%)	2 (3.4%)	
2 to > 6 times per day	3 (1.1%)	2 (1.0%)	1 (1.7%)	
Processed meats				0.652
≤ three times per month	231 (86.5%)	181 (87.0%)	50 (84.7%)	
1 to 6 times per week	36 (13.5%)	27 (13.0%)	9 (15.3%)	
White bread (slice)				0.002
≤3 times per month	75 (28.1%)	62 (29.8%)	13 (22.0%)	
1 to 6 times per week	136 (50.9%)	113 (54.3%)	23 (39.0%)	
Once daily	30 (11.2%)	17 (8.2%)	13 (22.0%)	
2 to > 6 times per day	26 (9.7%)	16 (7.7%)	10 (16.9%)	
Brown bread (slice)				0.252
≤3 times per month	87 (32.6%)	67 (32.2%)	20 (33.9%)	
1 to 6 times per week	137 (51.3%)	111 (53.4%)	26 (44.1%)	
Once daily	24 (9.0%)	15 (7.2%)	9 (15.3%)	
2 to > 6 times per day	19 (7.1%)	15 (7.2%)	4 (6.8%)	
Rice or pasta (cup amount)				<0.001
≤3 times per month	40 (15.0%)	35 (16.8%)	5 (8.5%)	
1 to 6 times per week	169 (63.3%)	143 (68.8%)	26 (44.1%)	
Once daily	43 (16.1%)	22 (10.6%)	21 (35.6%)	
2 to > 6 times per day	15 (5.6%)	8 (3.8%)	7 (11.9%)	
Dairy products				0.099
≤3 times per month	43 (16.1%)	35 (16.8%)	8 (13.6%)	
1 to 6 times per week	157 (58.8%)	128 (61.5%)	29 (49.2%)	
Once daily	48 (18.0%)	33 (15.9%)	15 (25.4%)	
2 to > 6 times per day	19 (7.1%)	12 (5.8%)	7 (11.9%)	
Dairy products containing beneficial bacteria				0.230
≤3 times per month	125 (46.8%)	93 (44.7%)	32 (54.2%)	
1 to 6 times per week	120 (44.9%)	98 (47.1%)	22 (37.3%)	
Once daily	16 (6.0%)	11 (5.3%)	5 (8.5%)	
2 to > 6 times per day	6 (2.2%)	6 (2.9%)	0 (0.0%)	
Citrus fruits				0.222
≤3 times per month	101 (37.8%)	76 (36.5%)	25 (42.4%)	
1 to 6 times per week	147 (55.1%)	118 (56.7%)	29 (49.2%)	
Once daily	13 (4.9%)	8 (3.8%)	5 (8.5%)	
2 to > 6 times per day	6 (2.2%)	6 (2.9%)	0 (0.0%)	
Non-acidic fruits				0.014
≤3 times per month	74 (27.7%)	58 (27.9%)	16 (27.1%)	
1 to 6 times per week	168 (62.9%)	135 (64.9%)	33 (55.9%)	
Once daily	16 (6.0%)	7 (3.4%)	9 (15.3%)	
2 to > 6 times per day	9 (3.4%)	8 (3.8%)	1 (1.7%)	
Leafy green vegetables				0.007
≤2 times per month	81 (30.3%)	69 (33.2%)	12 (20.3%)	
1 to 6 times per week	161 (60.3%)	126 (60.6%)	35 (59.3%)	
Once daily	19 (7.1%)	10 (4.8%)	9 (15.3%)	
2 to > 6 times per day	6 (2.2%)	3 (1.4%)	3 (5.1%)	
Vegetables				0.001
≤3 times per month	47 (17.6%)	35 (16.8%)	12 (20.3%)	
1 to 6 times per week	180 (67.4%)	151 (72.6%)	29 (49.2%)	
Once daily	28 (10.5%)	15 (7.2%)	13 (22.0%)	
2 to > 6 times per day	12 (4.5%)	7 (3.4%)	5 (8.5%)	
Nuts (fried/roasted)				0.047
≤3 times per month	159 (59.6%)	127 (61.1%)	32 (54.2%)	
1 to 6 times per week	99 (37.1%)	77 (37.0%)	22 (37.3%)	
Once daily	8 (3.0%)	3 (1.4%)	5 (8.5%)	
2 to > 6 times per day	1 (0.4%)	1 (0.5%)	0 (0.0%)	
Raw nuts				0.022
≤3 times per month	150 (56.2%)	119 (57.2%)	31 (52.5%)	
1 to 6 times per week	105 (39.3%)	84 (40.4%)	21 (35.6%)	
Once daily	10 (3.7%)	4 (1.9%)	6 (10.2%)	
2 to > 6 times per day	2 (0.7%)	1 (0.5%)	1 (1.7%)	
Spicy food				>0.999
≤3 times per month	127 (47.6%)	99 (47.6%)	28 (47.5%)	
1 to 6 times per week	107 (40.1%)	83 (39.9%)	24 (40.7%)	
Once daily	20 (7.5%)	16 (7.7%)	4 (6.8%)	
2 to > 6 times per day	13 (4.9%)	10 (4.8%)	3 (5.1%)	
Fried foods (dish)				0.482
≤3 times per month	112 (41.9%)	89 (42.8%)	23 (39.0%)	
1 to 6 times per week	136 (50.9%)	105 (50.5%)	31 (52.5%)	
Once daily	10 (3.7%)	6 (2.9%)	4 (6.8%)	
2 to > 6 times per day	9 (3.4%)	8 (3.8%)	1 (1.7%)	
Coffee (cup)				0.041
≤3 times per month	36 (13.5%)	24 (11.5%)	12 (20.3%)	
1 to 6 times per week	133 (49.8%)	113 (54.3%)	20 (33.9%)	
Once daily	53 (19.9%)	39 (18.8%)	14 (23.7%)	
2 to > 6 times per day	45 (16.9%)	32 (15.4%)	13 (22.0%)	
Tea (cup)				0.195
≤3 times per month	49 (18.4%)	36 (17.3%)	13 (22.0%)	
1 to 6 times per week	137 (51.3%)	114 (54.8%)	23 (39.0%)	
Once daily	41 (15.4%)	29 (13.9%)	12 (20.3%)	
2 to > 6 times per day	40 (15.0%)	29 (13.9%)	11 (18.6%)	
Sweets				0.347
≤3 times per month	71 (26.6%)	57 (27.4%)	14 (23.7%)	
1 to 6 times per week	153 (57.3%)	118 (56.7%)	35 (59.3%)	
Once daily	29 (10.9%)	20 (9.6%)	9 (15.3%)	
2 to > 6 times per day	14 (5.2%)	13 (6.3%)	1 (1.7%)	
Soft drinks or energy drinks (can)				0.455
≤3 times per month	126 (47.2%)	103 (49.5%)	23 (39.0%)	
1 to 6 times per week	115 (43.1%)	85 (40.9%)	30 (50.8%)	
Once daily	19 (7.1%)	14 (6.7%)	5 (8.5%)	
2 to > 6 times per day	7 (2.6%)	6 (2.9%)	1 (1.7%)	

Patterns of food preparation and the intake of vitamins and nutritional supplements

The prevalence of SD was significantly higher among participants who used butter for frying (20.3% n=12 vs 4.8% n=10 for those without SD, (p=0.003). Additionally, the proportion of participants with SD who consumed most of the visible fat in meat was significantly higher (16.9% n=10) than that of subjects without SD (6.7% n=14, p=0.049). The consumption of vitamin D was significantly higher among subjects with SD (60.0% n=18 vs. 33.8% n=25 among those without SD, p=0.014), whereas a significantly lower proportion of iron consumption was observed among SD patients compared to their peers without the disease (3.3% n=1 vs. 20.3% n=15, respectively, p=0.035) Table 3 goes into more details.

**Table 3:** Patterns of food preparation and the intake of vitamins and nutritional supplements

Characteristic	Overall, N=267	No, n=208	Yes, n=59	p-value
Cooking method used most often				0.053
Frying	150 (56.2%)	110 (52.9%)	40 (67.8%)	
Grilling	0 (0.0%)	0 (0.0%)	0 (0.0%)	
Poaching	117 (43.8%)	98 (47.1%)	19 (32.2%)	
Type of fat used for frying most often				0.003
Butter	22 (8.2%)	10 (4.8%)	12 (20.3%)	
Ghee	4 (1.5%)	3 (1.4%)	1 (1.7%)	
Vegetable oil	164 (61.4%)	135 (64.9%)	29 (49.2%)	
Olive oil	77 (28.8%)	60 (28.8%)	17 (28.8%)	
What do you do with the visible fat on meat?				0.049
I do not eat meat	20 (7.5%)	14 (6.7%)	6 (10.2%)	
I avoid the visible fat	179 (67.0%)	142 (68.3%)	37 (62.7%)	
I eat most of the visible fat	24 (9.0%)	14 (6.7%)	10 (16.9%)	
I eat some of the visible fat	44 (16.5%)	38 (18.3%)	6 (10.2%)	
How much salt do you add to your food when cooking or at the dinner table?				0.698
I do not take salt at all	4 (1.5%)	3 (1.4%)	1 (1.7%)	
Rarely	26 (9.7%)	21 (10.1%)	5 (8.5%)	
Sometimes	53 (19.9%)	39 (18.8%)	14 (23.7%)	
Usually	58 (21.7%)	43 (20.7%)	15 (25.4%)	
Always	126 (47.2%)	102 (49.0%)	24 (40.7%)	
Have you taken any vitamins, minerals, fish oils, fiber, or nutritional supplements in the past year?				0.018
No	158 (59.2%)	131 (63.0%)	27 (45.8%)	
Yes	109 (40.8%)	77 (37.0%)	32 (54.2%)	
How often do you take vitamins, minerals, fish oils, or nutritional supplements?				0.997
Never or less than once a month	16 (14.7%)	12 (15.6%)	4 (12.5%)	
1-3 times a month	19 (17.4%)	13 (16.9%)	6 (18.8%)	
Once a week	19 (17.4%)	13 (16.9%)	6 (18.8%)	
Once daily	46 (42.2%)	31 (40.3%)	15 (46.9%)	
2-3 daily	5 (4.6%)	4 (5.2%)	1 (3.1%)	
Vitamin D				0.014
No	61 (58.7%)	49 (66.2%)	12 (40.0%)	
Yes	43 (41.3%)	25 (33.8%)	18 (60.0%)	
Vitamin B				0.071
No	100 (96.2%)	73 (98.6%)	27 (90.0%)	
Yes	4 (3.8%)	1 (1.4%)	3 (10.0%)	
Omega 3				0.211
No	78 (75.0%)	58 (78.4%)	20 (66.7%)	
Yes	26 (25.0%)	16 (21.6%)	10 (33.3%)	
Multivitamin				0.564
No	87 (83.7%)	63 (85.1%)	24 (80.0%)	
Yes	17 (16.3%)	11 (14.9%)	6 (20.0%)	
Iron				0.035
No	88 (84.6%)	59 (79.7%)	29 (96.7%)	
Yes	16 (15.4%)	15 (20.3%)	1 (3.3%)	
Zinc				0.322
No	100 (96.2%)	70 (94.6%)	30 (100.0%)	
Yes	4 (3.8%)	4 (5.4%)	0 (0.0%)	
Vitamin E				>0.999
No	103 (99.0%)	73 (98.6%)	30 (100.0%)	
Yes	1 (1.0%)	1 (1.4%)	0 (0.0%)	
Vitamin C				>0.999
No	93 (89.4%)	66 (89.2%)	27 (90.0%)	
Yes	11 (10.6%)	8 (10.8%)	3 (10.0%)	
Magnesium				>0.999
No	102 (98.1%)	72 (97.3%)	30 (100.0%)	
Yes	2 (1.9%)	2 (2.7%)	0 (0.0%)	
Selenium				>0.999
No	103 (99.0%)	73 (98.6%)	30 (100.0%)	
Yes	1 (1.0%)	1 (1.4%)	0 (0.0%)	
Calcium				0.433
No	96 (92.3%)	67 (90.5%)	29 (96.7%)	
Yes	8 (7.7%)	7 (9.5%)	1 (3.3%)	

Characteristics of seborrheic dermatitis and the effect of food type on the disease

The majority of SD respondents (n=59) reported their disease severity as moderate, accounting for 20 individuals (45.8%). Less than half of them suffered from skin conditions other than SD. Among the respondents, 12 individuals (20.3%) indicated a positive family history of the condition (Table 4). The table also provides information on flare-ups or worsening of SD in response to certain food types. Notably, 29 (49.2%) participants reported no flare-ups with any food type. On the other hand, the following food types were commonly associated with SD exacerbation: spicy food (16.9% n=10), sweets (16.9% n=10), fried food (13.5% n=8), dairy products (11.9% n=7), and citrus fruits (10.2% n=6). In contrast, citrus fruits, leafy green vegetables (8.5% n=5 for each), and the other types of vegetables (6.8% n=4) were frequently observed with SD improvement.

**Table 4:** Characteristics of seborrheic dermatitis and the effect of food type on the disease SD - seborrheic dermatitis

Characteristic	n=59
Do you suffer from any skin diseases other than seborrheic dermatitis? Such as eczema, psoriasis, lichen planus?	25 (42.4%)
How severe was the disease when you were diagnosed and before any treatments began?	
Mild	14 (23.7%)
Moderate	27 (45.8%)
Severe	18 (30.5%)
Do you have relatives who have been diagnosed with seborrheic dermatitis?	
No	17 (28.8%)
Yes	12 (20.3%)
I do not know	30 (50.8%)
Flare-ups or worsening of SD with certain food types?	
No	29 (49.2%)
Yes:	30 (50.8%)
Sweets	10 (16.9%)
Spicy foods	10 (16.9%)
Fried Food	8 (13.5%)
Dairy products	7 (11.9%)
Seafood	6 (10.2%)
Nuts (fried/roasted)	6 (10.2%)
Citrus fruits	6 (10.2%)
Soft drinks or energy drinks	4 (6.8%)
Raw nuts	4 (6.8%)
White bread	3 (5%)
Processed meat	3 (5%)
Vegetables	2 (3.4%)
Red meat	2 (3.4%)
Coffee	2 (3.4%)
Chicken	2 (3.4%)
Rice or pasta	1 (1.7%)
Other: ketchup	1 (1.7%)
Eggs	1 (1.7%)
Improvement of SD symptoms with certain food types?	
No	47 (79.6%)
Yes:	12 (20%)
Citrus fruits	5 (8.5%)
Leafy green vegetables	5 (8.5%)
Vegetables	4 (6.8%)
Non-acidic fruits	3 (5.1%)
Brown bread	2 (3.4%)
Raw nuts	2 (3.4%)
Dairy products containing beneficial bacteria	2 (3.4%)
Chicken	1 (1.7%)
Rice or pasta	1 (1.7%)
Coffee	1 (1.7%)
Sweets	1 (1.7%)
Other: keto diet	1 (1.7%)

## Discussion

Our results have shown that consumption of simple carbohydrates, such as white bread, rice, and pasta, was significantly more prevalent amongst those diagnosed with SD. This anticipated outcome might be explained by the hormone IGF-1, which is known to be increased with carbohydrate intake. IGF-1 exerts its effects on sebaceous glands by stimulating their proliferation and increasing their production of sebum. Not unlike acne, SD is therefore expected to occur in those with a high frequency of carbohydrate intake. Indeed, a study compared IGF-1 levels in SD patients compared to controls. Not only was IGF-1 significantly higher in SD patients compared to controls (p=0.045), but those within the severe SD subcategory also demonstrated a higher median IGF-1 compared to the mild and moderate cases of SD (p=0.005) [10].

In addition, there was a significant association between the frequency of consuming non-acidic fruits, leafy green vegetables, roasted or fried nuts, raw nuts, and coffee in those with an SD diagnosis compared to the healthy group. A similar finding was also reported by Tamer, who indicated that the consumption of vegetables was significantly more frequent in patients with SD compared to controls (p=0.04), although no specifications regarding the types of vegetables inquired were mentioned. Despite their findings, the study done by Tamer ultimately concluded that diet is not associated with an increased risk of SD [11]. It is an unexpected finding that the consumption of nuts, whether fried, roasted, or raw, was seen in favor of the SD group, given the fact that nuts are known to contain polyphenols and vitamin E that induce an antioxidant effect and promote antiinflammation [12].

Although consumption of non-acidic fruits was more common within our SD cohort compared to the healthy controls with a statistically significant difference, this is in contrast to the results published by a large cross-sectional study performed by Sanders et al. [13], in which 4,379 participants were included with 636 (14.5%) of those participants diagnosed with SD. They have discovered that a fruit-based diet showed an up to 25% decreased risk of SD (p=0.03). In that same study, they also found that a Western diet consisting of processed meat, potatoes, and alcohol was associated with a 47% higher risk of SD (p=0.03), but only in female patients. Our study, however, did not reproduce such an association.

In the next part of the survey, we asked the patients' point of view on what flares or relieves their disease. Almost half of them did not notice any relationship between their diet and skin disease. The other half, however, have responded that spicy food, fried food, sweets, dairy products, and citrus fruits commonly exacerbated their symptoms. It is interesting that sweets containing high amounts of sugar were reported to be a disease aggravator by the SD cohort. In the FFQ, no difference was seen regarding the consumption of sweets between the cases and controls, yet in a different study, the total intake of sugar was significantly higher among the SD group (p<0.01) compared to the controls [14]. However, the reason for this association is not clear, especially when hyperinsulinemia as a cause of SD has been disproven by Dowlati and colleagues [15].

On the other hand, citrus fruits have also been reported by some patients to improve their symptoms, along with leafy green vegetables and other types of vegetables. While citrus fruits showed paradoxical results, this is perhaps due to other factors that play a confounding role between diet and SD, such as genetics, and since the results are patient-reported and based on their observations of what type of food relieved or flared up their SD, contradictions are possible. These results, however, are consistent with the findings published by Sanders, in which a fruit-based diet decreased the risk of SD, perhaps due to the antioxidant effect of fruits and vegetables, as well as the psoralen found in citrus fruits, which increases the skin sensitivity to UV radiation improving the symptoms of SD by suppressing *Malassezia* yeast growth as observed during the summer months in which SD is in its latency period [13]. Leafy green vegetables are also known to be rich in vitamin A, in which its active metabolite, retinoic acid, plays a role in suppressing sebum production, as is exploited in acne treatment. An inverse association between serum vitamin A and skin sebum amount was discovered by Boelsma et al. [16], which might explain why our SD cohort felt skin improvement when consuming vegetables.

The next section of the survey was concerned with the different variables of food preparation. The type of fat used for frying caused significant differences in the prevalence of SD; participants who responded to using butter for frying in most of their meals, as well as consuming most of the visible fat in meat, were significantly among the SD group. The excessive intake of trans or saturated fats can fuel systemic inflammation and worsen skin conditions, as is seen with another papulosquamous disease known as psoriasis [17]. Unlike our results, Sanders et al. found no association between a fat-rich diet and the risk of SD [11]. However, another noteworthy study identified that patients with SD had significantly lower levels of essential and unsaturated fatty acids compared to healthy controls (p<0.001) [18].

Interestingly, the prevalence of SD was higher amongst participants ingesting vitamin D supplements. We hypothesize that this is due to their vitamin D deficiency rather than the cholecalciferol supplements. This is supported by a case-control trial comparing the level of 25-hydroxyvitamin D in patients diagnosed with SD to healthy controls, and they've established that serum vitamin D was significantly lower in the SD group (p=0.007) and a low level was also associated with an increased severity of scalp SD (P= 0.003) [19]. Another study investigated the effects of Vitamin D supplementation on recurrences of SD in 32 patients and found that 65% of them had a reduction in the number of disease recurrences during treatment, yet 12.5% suffered an increase in the frequency of disease attacks [20].

The relationship between diet and skin disease is a non-linear and complex one, with many influencing factors. Emerging research is investigating the gut-skin axis, in which the interaction between gut microbes, immunity, and metabolic properties is intimately linked with skin health [21]. Although our results showed no significant difference between the cases and controls regarding consumption of dairy products containing probiotic bacteria, a double-blinded, randomized study has demonstrated that after a washout period, the ingestion of the probiotic *Lactobacillus paracasei* for 56 days with no dandruff treatment applied other than a mild shampoo, was associated with an improvement of scalp erythema as well as a decrease in scalp seborrhea and dandruff seen in scalp SD with a statistical significant difference (p<0.05). It worked by improving the skin barrier and restoring the balance of scalp microbiota, the disturbance of which is the root cause of SD development [22].

Specific dietary triggers for SD can be tested by implementing an elimination diet trial. However, we cannot recommend eliminating certain types of food without further corroborating evidence. Therefore, consuming a healthy, well-balanced diet should be advised to all patients. A Mediterranean diet, for example, known for its antioxidant effect, consists of olive oil, nuts, vegetables, fruits, and seafood while limiting the consumption of processed meat, added sugar, and refined grains. Nevertheless, referral to a clinical dietitian is recommended for a dietary intervention tailored for each individual patient by considering their food allergies, medical conditions, and personal goals.

The findings of this study are subject to a few limitations. The first being is the small sample size due to overall poor response to the survey. Of over a thousand potential participants diagnosed with SD by a dermatologist who were sent invitations, only 85 responded. This can be due to a lack of incentive to fill out the research or a lack of awareness of the importance of research and its significant effect on the medical field. Another limitation is the subjective, self-reported, and retrospective nature of our data, which can lead to recall bias. Finally, due to the observational design of this study, many lifestyle factors could not be controlled, which leaves the possibility of residual confounding.

## Conclusions

Several dietary factors have been associated with SD in our cohort, indicating that diet may affect the course of the disease. These include white bread, rice, pasta, non-acidic fruits, leafy green vegetables, roasted or fried nuts, raw nuts, and coffee. Citrus fruits and vegetables were reported to relieve the symptoms of SD by our cohort. On the other hand, spicy food, sweets, fried food, dairy products, and citrus fruits were among the disease aggravators. Before dietary restriction can be applied, further evidence is needed to substantiate our results. We advise future researchers to include a prospective daily diet diary to decrease memory bias, modify participants' diets with the help of a clinical dietitian, as well as frequent clinical visits to a dermatologist so that changes in disease severity can be objectively noted. We also suggest going beyond the limitations of the FFQ and diving deeper into investigating the gut-skin axis by including investigational methods such as laboratory tests and histopathology to unravel the exact relationship between diet and dermatological diseases.
